# Effects of Src Kinase Inhibition on Expression of Protein Tyrosine Phosphatase 1B after Brain Hypoxia in a Piglet Animal Model

**DOI:** 10.1155/2017/2810295

**Published:** 2017-05-23

**Authors:** Dimitrios Angelis, Maria Delivoria-Papadopoulos

**Affiliations:** ^1^Department of Pediatrics, Texas Tech University-HSC at the Permian Basin, Odessa, TX, USA; ^2^Department of Pediatrics, Drexel University and St. Christopher's Hospital for Children, Philadelphia, PA, USA

## Abstract

**Background:**

Protein tyrosine phosphatases (PTPs) in conjunction with protein tyrosine kinases (PTKs) regulate cellular processes by posttranslational modifications of signal transduction proteins. PTP nonreceptor type 1B (PTP-1B) is an enzyme of the PTP family. We have previously shown that hypoxia induces an increase in activation of a class of nonreceptor PTK, the Src kinases. In the present study, we investigated the changes that occur in the expression of PTP-1B in the cytosolic component of the brain of newborn piglets acutely after hypoxia as well as long term for up to 2 weeks.

**Methods:**

Newborn piglets were divided into groups: normoxia, hypoxia, hypoxia followed by 1 day and 15 days in FiO_2_ 0.21, and hypoxia pretreated with Src kinase inhibitor PP2, prior to hypoxia followed by 1 day and 15 days. Hypoxia was achieved by providing 7% FiO_2_ for 1 hour and PTP-1B expression was measured via immunoblotting.

**Results:**

PTP-1B increased posthypoxia by about 30% and persisted for 2 weeks while Src kinase inhibition attenuated the expected PTP-1B-increased expression.

**Conclusions:**

Our study suggests that Src kinase mediates a hypoxia-induced increased PTP-1B expression.

## 1. Introduction

Protein tyrosine phosphatases (PTPs) are a superfamily of receptor-like proteins that act as important modulators of cellular functions. PTPs contain a 250-amino acid catalytic domain specialized against phosphorylated tyrosine residues [1]. PTP nonreceptor type 1B (PTP-1B) is the most well-known and studied member of this family. PTP-1B is ubiquitous in tissues and regulates several metabolic processes, including the function of receptor tyrosine kinases (RTKs) such as the platelet-derived growth factor (PDGF) and epidermal growth factor receptor (EGFR). PTP-1B antagonizes the action of adipose-secreting hormone leptin by directly dephosphorylating the leptin receptor-associated Janus kinase 2 [2, 3]. PTB-1B is also involved in glucose homeostasis by regulating the insulin receptor (IR) [4]. In the central nervous system, PTP-1B affects cellular energy balance, axonal arborization, neurite outgrowth, signaling, and neuronal plasticity. PTP-1B regulates biochemical processes associated with the endoplasmic reticulum (ER) stress. The pathogenesis of several neurodegenerative diseases, such as Alzheimer's and Parkinson's diseases [5], has been recently attributed to ER stress, and PTP-1B inhibition has shown neuroprotective effects [6]. PTP-1B is also a physiological regulator of brain-derived neurotrophic factor (BDNF) signaling in neurons. Decreased BDNF signaling is linked to energy balance dysregulation, inflammation, and obesity [7, 8].

A key intracellular nonreceptor cytosolic kinase is the Src family kinase. Src kinases are expressed in the central nervous system via their neuronal subtypes (Src, Fyn, and Yes) and regulate cellular movement, membrane channel activation, and neuronal growth [9]. Brain hypoxia activates Src kinase, and its inhibition with a specialized inhibitor, PP2, was shown to affect several components of the apoptotic and pyroptotic pathways via presumptive tyrosine phosphorylation of key components. Src kinase regulates the immune system via the formation of specific platforms of innate immunity, the inflammasomes. The inflammasomes detect intracellular or extracellular danger signals, become activated, and trigger cysteine protease caspase-1-dependent processing of the proinflammatory cytokine IL-1*β* in the macrophage. Bacterial or viral pathogens can activate the NLR3 inflammasome/caspase-1/IL-1*β* pathway via Src kinase-mediated mechanisms [10]. Src kinase has been also shown to affect caspase-1 activity in hippocampal neurons derived from mice. Src kinase deficient neurons exhibit significantly increased caspase-1 activity [11].

PTPs in conjunction with protein tyrosine kinases (PTKs) regulate opposing cellular processes by maintaining a balance between phosphorylation and dephosphorylation of key enzymatic pathways and affecting the activation status of several intracellular components. This interaction is achieved to a certain degree, by specific Src homology domains (such as SH2 and SH3) that characterize PTPs and can associate with PTKs. By their competing roles, PTPs and PTKs assure the correct interpretation of extracellular signals into specified cellular behaviors [12]. The role of Src kinase in the pathway of PTP-1B after brain hypoxia has not been adequately investigated. The aim of this study was to characterize the changes that occur in PTP-1B expression after 1 hour of the induced hypoxic insult (acute phase), at 1 day as well as at 15 days after hypoxia (posthypoxia phase) in the brain of term newborn piglets. We investigated if hypoxia-induced changes in PTP-1B expression are mediated by Src kinase.

## 2. Methods and Materials

### 2.1. Experimental Protocol

The experimental protocol was approved by the Institutional Animal Care and Use Committee of Drexel University (IACUC Approval Number: 200491). All procedures were performed under anesthesia, and all efforts were made to minimize pain and suffering.

**Figure 1:**
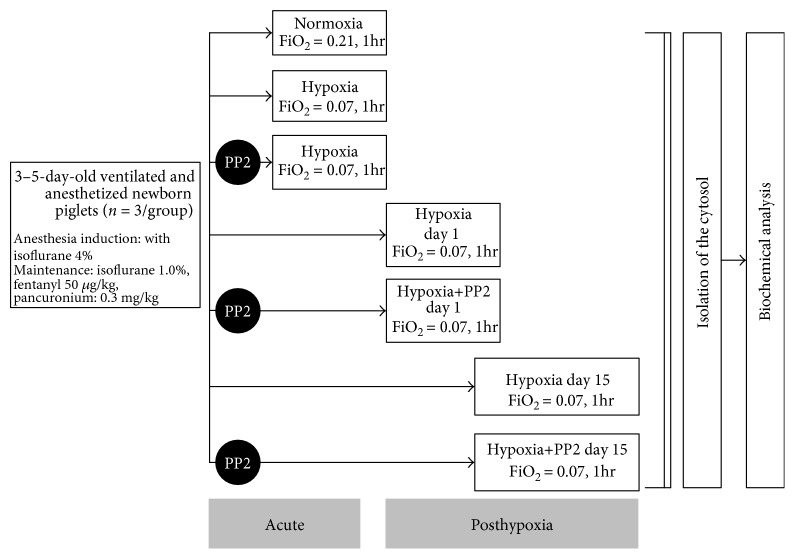
Experimental protocol.

The experiments were conducted on anesthetized, ventilated 3–5 day old term newborn piglets. The piglets were allocated into the following groups designated as “acute phase”: normoxic (Nx), hypoxic (Hx), and hypoxic pretreated with Src kinase inhibitor PP2 (Hx+PP2). Piglets that were left to recover after hypoxia were followed, under normoxic conditions, for either 1 or 15 days designated as “posthypoxia phase” (Figure 1). There were three piglets in each group. For induction of anesthesia, isoflurane was used, while for maintenance, nitrous oxide and fentanyl (50 *μ*g/kg, IV) was used. Conventional mechanical ventilation (pressure control mode) was used in all piglets with adequate adjustments in the settings so as to maintain normocarbia. After intubation, paralysis was implemented with pancuronium (0.3 mg/kg, IV). Body temperature was maintained at 38.5–39.5°C. In piglets that were assigned to hypoxia, FiO_2_ was decreased to 7% for one hour, while the piglets assigned to the normoxia group were ventilated at FiO_2_ = 21% for one hour. Piglets assigned to the Hx+PP2 group received a selective Src kinase inhibitor (PP2, 1 mg/kg, IV), 30 min prior to the hypoxic exposure. At the end of the study, cerebral cortex was removed and homogenized. The “posthypoxia” animals were anesthetized and their brain was removed on day 1 and 15. After the brain removal, one gram of cerebral cortical tissue was homogenized by a Dounce-type glass homogenizer. The homogenized tissue was centrifuged at 1000 ×g for ten minutes at 4°C and then again at 15,000 ×g for 1 hour at 4°C. Subsequently, the supernatant was centrifuged again at 100,000 ×g for 60 minutes at 4°C to obtain the cytosolic fraction.

Further biochemical analysis was performed as described in methods. During this procedure, piglets that undergo hypoxia, become acidotic, and develop hypotension with mean arterial pressure at the limits of loss of autoregulation. This experimental model of combined brain hypoxia ischemia as well as the details in changes of blood pressure and acid-base status of the ventilated piglets is previously described [13].

### 2.2. Enzymatic Determination of ATP and Phosphocreatine (PCr)

ATP and PCr concentrations were determined by spectrophotometry using the Lamprecht method [14] as previously described [15]. In summary, the deproteinized cortical homogenate was centrifuged at 4000 ×g for 5 minutes at 4°C. Aliquots of the supernatant were neutralized with KOH-K_2_CO_3_ and centrifuged at 2000 ×g for 5 minutes at 4°C. ATP and PCr concentrations were determined in a 1 ml volume containing buffer (50 mM triethanolamine, 5 mM MgCl_2_, 1 mM EDTA, 2 mM glucose, pH 7.6), 400 *μ*l of neutralized 2000 ×g supernatant, and 20 *μ*l NADP. Readings were made every 5 minutes after the addition of 10 *μ*l hexokinase until a steady state was reached. The ATP concentration was calculated from the increase in absorbance at 340 nm during 20 minutes after the addition of hexokinase. PCr concentration was calculated from the increase in absorbance at 340 nm after the addition of creatine kinase.

### 2.3. PTP-1B Expression

Immunoblotting was performed as previously described by Ashraf et al. [16]. Cytosolic proteins were incubated with primary polyclonal antibodies for PTP-1B (Santa Cruz Biotech, Santa Cruz, CA). Equal protein loading was assured with the following 2 methods: (a) by estimating equal amounts of loading protein and (b) by using *β*-actin to verify homogenous loading transfer. Blots were incubated with a polyclonal rabbit antibody against PTP-1B with a dilution of 1 : 1000 in PBS/1% serum at room temperature. PBS was used for washing the slides, and subsequently, those were incubated with a biotin-conjugated secondary antibody (Jackson Labs). Immunocomplexes were detected by assessing the emission of light during the horse radish peroxides and hydrogen peroxide-catalyzed oxidation of luminol with the use of enhanced chemiluminescence (ECL) detection system (GE Healthcare, USA). The protein density was expressed as absorbance (OD × mm^2^) using analysis by imaging densitometry (GS-700 densitometer, UK).

The data were analyzed using Sigma Plot version 13.0 (San Jose, Ca). Analysis of variance of repeated measures was used for comparisons between and within the 3 groups (normoxia, hypoxia, and hypoxia+PP2) at each time point (0, 1 h, 1 D, and 15 D). For post hoc analysis, the Fisher exact test was used. The data are presented as mean ± standard deviation (SD). A *P* value of 0.05 was considered significant.

## 3. Results

### 3.1. ATP and Phosphocreatine (PCr) Levels

The ATP levels (*μ*moles/g brain) were Nx = 4.4 ± 0.4, Hx = 1.6 ± 0.3, and Hx+PP2 = 1.7 ± 0.4. The PCr levels (*μ*moles/g brain) were Nx = 3.5 ± 0.2, Hx = 1.3 ± 0.3, and Hx+PP2 = 1.2 ± 0.3. The hypoxic groups had decreased levels of cerebral high-energy phosphates when compared to the normoxia group (*P* < 0.05), demonstrating tissue hypoxia in the acute phase in both groups. Pretreatment with PP2 did not affect the levels of ATP and PCr that were measured following acute hypoxia (P NS).

### 3.2. PTP-1B Expression

**Figure 2:**
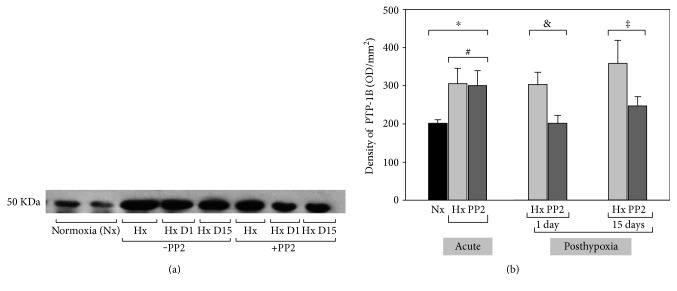
(a) Immunoblotting was performed with an antibody specific for the PTP-1B. One band was identified at 50 kDa corresponding to the active PTP-1B. Results are expressed as OD values (mean ± SD). In this figure, also, we include data from piglets that were left to recover after 1 hour of hypoxia (for 1 day and for 15 days). (b) There was an increased expression of PTP-1B acutely after hypoxia that was maintained up to 15 days. Pretreatment with a Src inhibitor (PP2) did not affect the expression of PTP-1B in the acute phase 1 hour after hypoxia but blocked the expression of PTP-1B at 1 day posthypoxia. There was a trend for decreased PTP-1B expression at 15 days in piglets that were pretreated with PP2, but this did not reach statistical significance. Acute groups: ^∗^*P* < 0.05 Hx and Hx+PP2 versus Nx. ^#^NS Hx versus Hx+PP2. Recovery 1 day: ^&^*P* < 0.05 Hx D1 versus Hx D1+PP2 and Nx. Recovery 15 days: ^‡^NS Hx D15 versus Hx D15+PP2 and Nx.

In Figure 2(a), an immunoblot of PTP-1B in the normoxia (Nx), hypoxia (Hx), and hypoxia pretreated with PP2 (Hx+PP2) is shown. In this figure, the “posthypoxia phase” groups either without or with PP2 are also shown (hypoxia 1 day posthypoxia, Hx D1; 15 days posthypoxia, Hx D15; pretreated with PP2 1 day posthypoxia, Hx D1+PP2; and pretreated with PP2 15 days posthypoxia, Hx D15+PP2). The molecular weight of PTP-1B was close to 50 kDa consistent with literature data. In the acute phase, the protein density (OD × mm^2^) of PTP-1B was 202 ± 8.9 in Nx, 305 ± 41 in Hx (versus Nx), and 300 ± 40 in Hx+PP2 (*P* < 0.05 versus Nx, NS versus Hx). During the posthypoxia phase day 1, the protein density of PTP-1B (Hx D1) was 303 ± 32 (*P* < 0.05 versus Nx, NS versus hypoxic groups) and 204 ± 19 in the PP2-treated hypoxic group, Hx D1+PP2 (*P* < 0.05 versus Hx, Hx D1, but NS versus Nx). During the posthypoxia phase day 15, the protein density of PTP-1B (Hx D15) was 360 ± 59 (*P* < 0.05 versus Nx, NS versus hypoxic groups) and 247 ± 25 in the PP2-treated hypoxic group, Hx D15+PP2 (P NS versus Nx or hypoxic groups). These data are shown in Figure 2(b). In this animal model, production of PTP-1B is increased after brain hypoxia, and administration of PP2 prior to the hypoxic insult attenuates the increase in the expression of the PTP-1B at day 1 posthypoxia, but not long term.

## 4. Discussion

In this study, we provide evidence that PTP-1B-increased expression is achieved early after brain hypoxia. This effect is preserved for 15 days. Under cellular stress, such as brain hypoxia/ischemia, PTP-1B activation status changes. There are different and competing cellular mechanisms that regulate PTP-1B activation. PTP-1B activation in brain samples, posthypoxia, increases in the cytosolic fraction of the cortical cells but decreases in the fraction derived from cellular membranes [17]. This possibly represents a localization effect of the PTP-1B as well as a difference in the status of enzymatic activation. PTP-1B exerts its function as an intracellular phosphatase on the cytoplasmic face of the endoplasmic reticulum [18]. A presumptive mechanism for activation of PTP-1B posthypoxia would be the presence of reactive oxygen species (ROS). ROS modulate the chemical environment of the catalytic site of PTP-1B [1, 19]. Oxidation of the active site inhibits PTP-1B activity. ROS functions as an intracellular messenger and activator of receptor tyrosine kinases [20]. An opposite effect would be the activation of neuroinflammation that follows hypoxia, which appears to increase PTP-1B expression [21]. The net effect of these opposing mechanisms in our study was an immediate increase in PTP-1B expression. It is possible that the initial increase of PTP-1B represents a “preformed” fraction of the molecule, while transcriptional changes and the effects of hypoxia via different complex molecular pathways take effect later in the course of hypoxia. Similar immediate effects were noted posthypoxia in several inflammation or apoptosis-associated proteins following this experimental method. Src kinase inhibition did not affect the levels of PTP-1B immediately after hypoxia, but later at 1 day. In this study, we did not assess the activity of PTP-1B. PTP-1B has a catalytic constant of ~2 × 10^3^ molecules/min which makes its interaction with molecules very brief and difficult to measure [22].

**Figure 3:**
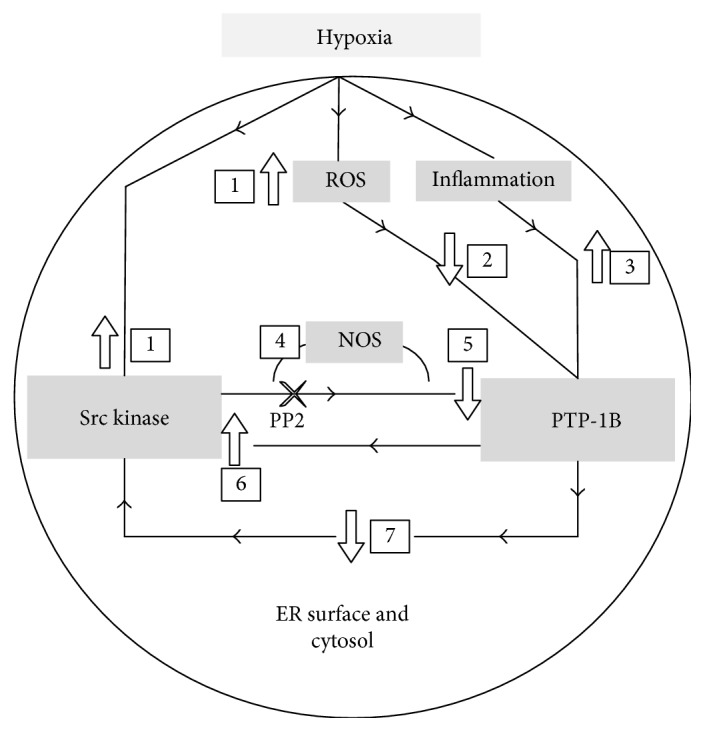
(1–3) Hypoxia induces PTP-1B and Src kinase. In this paradigm, ROS are shown as a mediator for this effect. Inflammatory pathways could also culminate to the activation of Src and PTP-1B. (4, 5) Src kinase phosphorylates and interferes with PTP-1B action. Use of PP2 (an Src kinase inhibitor) would ameliorate the effects of Src kinase and hence would inhibit PTP-1B production as shown in this study. Nitric oxide synthase might also interfere in this pathway. NO production was increased in cells overexpressing PTP-1B and treatment with PP2 significantly decreased its production. (6) Src kinase has a negative regulatory phosphorylation site (tyrosine-527). PTP-1B is found to dephosphorylate Src kinase at this residue. Tyr-527 occupies the carboxy-proximal area of Src and phosphorylation at this site has been shown to deactivate the enzyme. PTP-1B dephosphorylates and activates Src. (7) Under different circumstances, PTPs may dephosphorylate specific components of the Src kinase-associated complexes and lead to an actual disassembly of those and loss of their activity.

Src kinase inhibition prevented the observed PTP-1B rise posthypoxia. This effect was also seen to extend at the recovery phase although the results did not reach statistical significance. The findings of the current study could be explained by a possible interference of Src in the pathway of PTP-1B. Several phosphatases have been shown to dephosphorylate Src. PTP-1, SHP-1, and SHP-2 have all been shown to be involved in the regulation of Src in cancer cells [23] but have not been adequately investigated after hypoxia in neurons. Src kinase has a negative regulatory phosphorylation site (tyrosine-527). PTP-1B, when investigated in breast cancer cells, was found to dephosphorylate Src kinase at this residue [24]. Tyr-527 occupies the carboxy-proximal area of Src, and phosphorylation at this site has been shown to deem the enzyme inactive. The interaction between the phosphorylated Tyr-527 and the phosphorylated SH2 causes the “closing” of Src, and hence, the dephosphorylation at this site by PTP-1B would activate Src. This interaction might be quite different depending on the methodology followed. PTP-1B interacts also with a Src-homology 3 (SH3) domain which are present in components such as p130CAS and Src kinase [25, 26]. Tyrosine phosphorylation of FAK (a key component of the Src activation complex) facilitates the recruitment of several Src homology domains. Subsequently, the activated FAK/Src complex, facilitates the assembly and recruitment of other adaptor proteins such as the paxillin and p130CAS. Dephosphorylation of tyrosine residues results in the disassembly of the Src/FAK/paxillin complex. PTPs including PTP-1B might be involved in a negative feedback loop. In fibroblasts, PTP-1B has been shown to dephosphorylate FAK at Tyr-397, the primary Src kinase binding site [27]. A simple representation of this interaction is shown in Figure 3. The opposing effects of phosphatases on the Src kinase activation status are changed by the type of insult and underlying cellular function of the affected tissue. In this figure, Src and PTP-1B are shown to interact in a bidirectional manner.

The enzymatic interactions of Src and PTP-1B have as prerequisite the close proximity of those enzymatic pathways. Topographically, PTP-1B by having a small hydrophobic fragment at the C-terminus attaches to the ER with its catalytic center towards the cytoplasm. PTP-1B can also be released completely from the ER, under ER stress [28], although this effect has not been proved in the event of hypoxia. Src kinase components have been found at the ER, within focal adhesions and at areas of cellular attachments. FAK, Src, and paxillin (components of the Src kinase complexes) have also been described at areas of cellular interaction [29] and in close interaction with a variety of PTPs [30]. Information gathered mainly from experiments involving interaction between PTP-1B and RTKs (IR, PDGF, and EGFR) shows that PTP-1B can access its substrates via endocytosis, increased biosynthesis, or ER movement towards the plasma membrane at areas of cell-cell interaction. These are no direct evidence to which of the above mechanism is predominant in Src/PTP-1B interaction after brain hypoxia. It has been suggested though that the abovementioned differences in location account at least in part for substrate selection by PTPs.

We provide evidence that PTP-1B increases posthypoxia in the cortical homogenate of piglets. The role of PTP-1B in CNS has recently started to be elucidated. PTP-1B is a key regulator of BDNF/TrkB which helps neural connectivity by facilitating axonal growth [31]. PTP1B/TrkB interaction and TrkB receptor dephosphorylation occur after ligand-induced receptor internalization, as in other RTKs or via intracellular relocalization of a smaller cleaved, but active form of PTP-1B into the cytosol [32]. PTP-1B appears also to act as a regulator of neuroinflammation. Inflammatory responses after brain hypoxia include a variety of mechanisms such as the activation of the neuronal resident cells (macrophages and microglia) and/or inflammatory mediators, such as caspase-1/IL-1*β*, NO, and chemokines. Neuroinflammation contributes to cellular death acutely and long term. Src kinase has been shown to be involved in caspase-1/IL-1*β* pathway. In a mouse model, brain PTP-1B expression was found to increase after injection of LPS [21]. In this experimental methodology, Src kinase was found to be a substrate of PTP-1B. Src kinase activation was increased by LPS and Src activity was significantly inhibited by PTP-1B pretreatment. We have previously shown that pretreatment with a selective NOS inhibitor decreases the hypoxia-induced modification in the activity and expression of PTP [17] while in the study of Song et al., NO production was increased in cells overexpressing PTP-1B and treatment with PP2 significantly decreased its production [21].

## 5. Conclusions

Posthypoxia, Src kinase modulates several proapoptotic and apoptotic components (such as caspase-3, 9, and 8) as well as neuroinflammatory processes (caspase-1, IL-1). In this experiment, Src kinase inhibition prior to hypoxia resulted in attenuation of a previously increased expression of PTP-1B. This effect might represent the endpoint of an Src-mediated inhibition of PTP-1B via tyrosine phosphorylation. Our study suggests that Src kinase mediates hypoxia-induced increased PTP-1B expression.

## Abbreviations

CNS: Central nervous system
EGFR: Epidermal growth factor receptor
ER: Endoplasmic reticulum
FAK: Focal adhesion kinase
IR: Insulin receptor
NLR: NOD-like receptors
NO: Nitric oxide
NOS: Nitric oxide synthase
PDGF: Platelet-derived growth factor
PP2: 4-Amino-5-(4-chlorophenyl)-7-(t-butyl)pyrazolo[3, 4-d]pyrimidine
PTKs: Protein tyrosine kinases
PTPs: Protein tyrosine phosphatases
ROS: Reactive oxygen species
RTK: Receptor tyrosine kinases.

## Additional Points

*Highlights*. (1) Brain hypoxia induces apoptosis and neuroinflammation. (2) Src (a nonreceptor tyrosine kinase) activation is increased posthypoxia in neurons. (3) PTP-1B is a key phosphatase that controls metabolism via phosphorylation of several intracellular receptor tyrosine kinases. (4) There is increasing evidence that PTP-1B interacts and regulates Src kinase, but these effects have not been proved under brain hypoxia in neurons. (5) We provide evidence that PTP-1B-increased expression posthypoxia is attenuated after Src kinase inhibition.
